# Basal-like Phenotype Identifies Immunotherapy-Responsive Subset of HR+/HER2- Breast Cancer with Aggressive Clinical Behavior

**DOI:** 10.3390/cancers18182965

**Published:** 2026-09-14

**Authors:** Fangyu He, Yu Wu, Lu Pan, Keming Chen, Jiehua He, Jiabin Lu, Mei Li, Xue Chao, Rongzhen Luo, Xi Cai, Ziqing Zhao, Yuxuan Wu, Jinhui Zhang, Peng Sun

**Affiliations:** 1 State Key Laboratory of Oncology in South China, Collaborative Innovation Center for Cancer Medicine, Guangzhou 510060, China; hefy1@sysucc.org.cn (F.H.); wuyu2@sysucc.org.cn (Y.W.); panlu@sysucc.org.cn (L.P.); chenkm@sysucc.org.cn (K.C.); hejh@sysucc.org.cn (J.H.); lujb@sysucc.org.cn (J.L.); limei@sysucc.org.cn (M.L.); chaoxue@sysucc.org.cn (X.C.); luorzh@sysucc.org.cn (R.L.); caixi@sysucc.org.cn (X.C.); zhaoziqing@sysucc.org.cn (Z.Z.); wuyuxuan@sysucc.org.cn (Y.W.); 2 Department of Pathology, Sun Yat-Sen University Cancer Center, Guangzhou 510060, China; 3 Foshan Hospital of Traditional Chinese Medicine, Foshan 528099, China

**Keywords:** HR+/HER2- breast cancer, basal-like phenotype, Immune microenvironment, immunotherapy, prognosis

## Abstract

Background: While basal-like markers (BMs), particularly cytokeratin 5/6 (CK5/6) and epidermal growth factor receptor (EGFR), are traditionally associated with triple-negative breast cancer, their expression in HR+/HER2- tumors defines a clinically distinct subgroup with unique therapeutic vulnerabilities. We comprehensively characterized the clinicopathological features, immune microenvironment, and neoadjuvant immunotherapy response of BM-positive HR+/HER2- breast cancer. Methods: We analyzed 150 basal marker-positive (BM+) and 180 BM-negative HR+/HER2- breast cancer patients. Clinicopathological data and survival outcomes were assessed using Kaplan–Meier and Cox regression analyses. The immune microenvironment was characterized by stromal tumor-infiltrating lymphocytes (sTILs) and immunohistochemistry for immune markers (CD3, CD8, FOXP3, CXCL13, CD68, PD-1, PD-L1). An independent cohort of 53 BM+ patients receiving neoadjuvant chemotherapy with anti-PD-1/PD-L1 immunotherapy was evaluated for pathological complete response (pCR). Results: BM+ patients exhibited significantly more aggressive features: younger age at diagnosis, higher histological grade, increased necrosis, and lower hormone receptor expression. BM+ status independently predicted worse disease-free survival (HR = 1.96, *p* = 0.034) and overall survival (HR = 2.93, *p* = 0.037), with CK5/6 expression emerging as an independent prognostic factor for both endpoints. These tumors displayed an activated immune microenvironment with significantly higher sTILs, enhanced cytotoxic T-cell infiltration, and elevated PD-L1 expression. Remarkably, the neoadjuvant immunotherapy cohort achieved a pCR rate of 49.1%, comparable to triple-negative breast cancer rates and substantially exceeding historical HR+/HER2- benchmarks. Conclusions: The basal-like phenotype identifies a clinically aggressive subset of HR+/HER2- breast cancer with distinct immune-activated characteristics and remarkable immunotherapy response rates. These findings suggest that the BM status, particularly CK5/6 expression, may serve as a basis for clinicians to decide whether to administer immunotherapy to patients with HR+/HER2-breast cancer.

## Simple Summary

HR+/HER2- breast cancer represents 70% of all breast cancers but has shown limited benefit from immunotherapy, with pathological complete response (pCR) rates of only 24–30% compared to 58–66% in triple-negative breast cancer (TNBC). This resistance has been considered an immutable biological characteristic, leading to exclusion of most HR+/HER2- patients from immunotherapy strategies. However, emerging evidence suggests this subtype is biologically heterogeneous, with approximately 16–20% exhibiting basal-like features despite retaining hormone receptor expression. We present a clinically practical solution using standard immunohistochemistry to identify basal marker-positive (BM+) HR+/HER2- breast cancer. We demonstrate that BM+ tumors exhibit aggressive features and significantly worse survival. Crucially, these tumors display an activated immune microenvironment with higher stromal tumor-infiltrating lymphocytes (TILs), enhanced cytotoxic T-cell infiltration, and elevated PD-L1 expression. In an independent cohort of 53 BM+ patients receiving neoadjuvant immunotherapy, we achieved a remarkable pCR rate of 49.1%, nearly double the historical benchmark for unselected HR+/HER2- patients and approaching TNBC response rates.

## 1. Introduction

Hormone receptor (HR)-positive, human epidermal growth factor receptor 2 (HER2)-negative breast cancer (BC) represents the most prevalent molecular subtype, accounting for approximately 70% of all breast cancer cases [1,2]. While endocrine therapy has dramatically improved outcomes for most patients, a significant subset exhibits intrinsic or acquired resistance, leading to disease recurrence and mortality [3,4]. This clinical heterogeneity underscores a critical unmet need: identifying high-risk subpopulations that may benefit from alternative therapeutic strategies beyond conventional endocrine approaches.

Current clinical classification relies primarily on HR and HER2 status, yet this framework fails to capture the biological diversity within HR+/HER2- disease. Molecular profiling, such as Prediction Analysis of Microarray 50 (PAM50) classification, a gene-expression-based molecular subtyping system that sorts breast cancers into four intrinsic subtypes including Luminal A, Luminal B, HER2-enriched and basal-like by using 50 signature genes to guide prognosis and treatment selection, reveals that approximately 16–20% of HR+/HER2- tumors display basal-like phenotypes despite retaining hormone receptor expression [5,6]. These tumors co-express basal markers (BMs) such as cytokeratin 5/6 (CK5/6) and epidermal growth factor receptor (EGFR)—features traditionally associated with triple-negative breast cancer (TNBC). However, this biologically distinct subgroup remains unrecognized in routine clinical practice, potentially leading to suboptimal treatment selection.

The therapeutic implications of this oversight become particularly evident in the era of immunotherapy. While immune checkpoint inhibitors have revolutionized TNBC treatment achieving pathological complete response (pCR) rates of 58–66% in neoadjuvant settings [7,8,9,10,11,12], their efficacy in unselected HR+/HER2- patients remains modest, with pCR rates of only 24–30% [12,13,14]. This stark contrast raises a pivotal question: could the apparent resistance of HR+ breast cancer to immunotherapy actually stem from our failure to recognize BM-positive HR+/HER2- tumors as a distinct subgroup that might possess the immunological features necessary for robust treatment response, similar to their TNBC counterparts?

This study comprehensively characterizes the clinicopathological features, survival outcomes, and immune microenvironment of BM-positive HR+/HER2- BC to address this question. Furthermore, we evaluate the neoadjuvant immunotherapy response in an exploratory cohort of these patients. Our findings aim to provide evidence for integrating basal-like markers assessment into clinical decision-making for HR+/HER2- BC.

## 2. Materials and Methods

### 2.1. Study Design and Patient Cohorts

This retrospective study comprised two independent patient cohorts: (1) a primary cohort for comprehensive clinicopathological and prognostic analysis of basal marker-positive (BM+) versus basal marker-negative (BM-) HR+/HER2- breast cancer, and (2) an exploratory immunotherapy cohort evaluating neoadjuvant immunotherapy response in BM+ BC patients. The study was approved by the Institutional Review Board of Sun Yat-sen University Cancer Center (SYSUCC), with waiver of informed consent for retrospective analysis.

### 2.2. Primary Cohort: Clinicopathological and Survival Analysis

**Figure 1:**
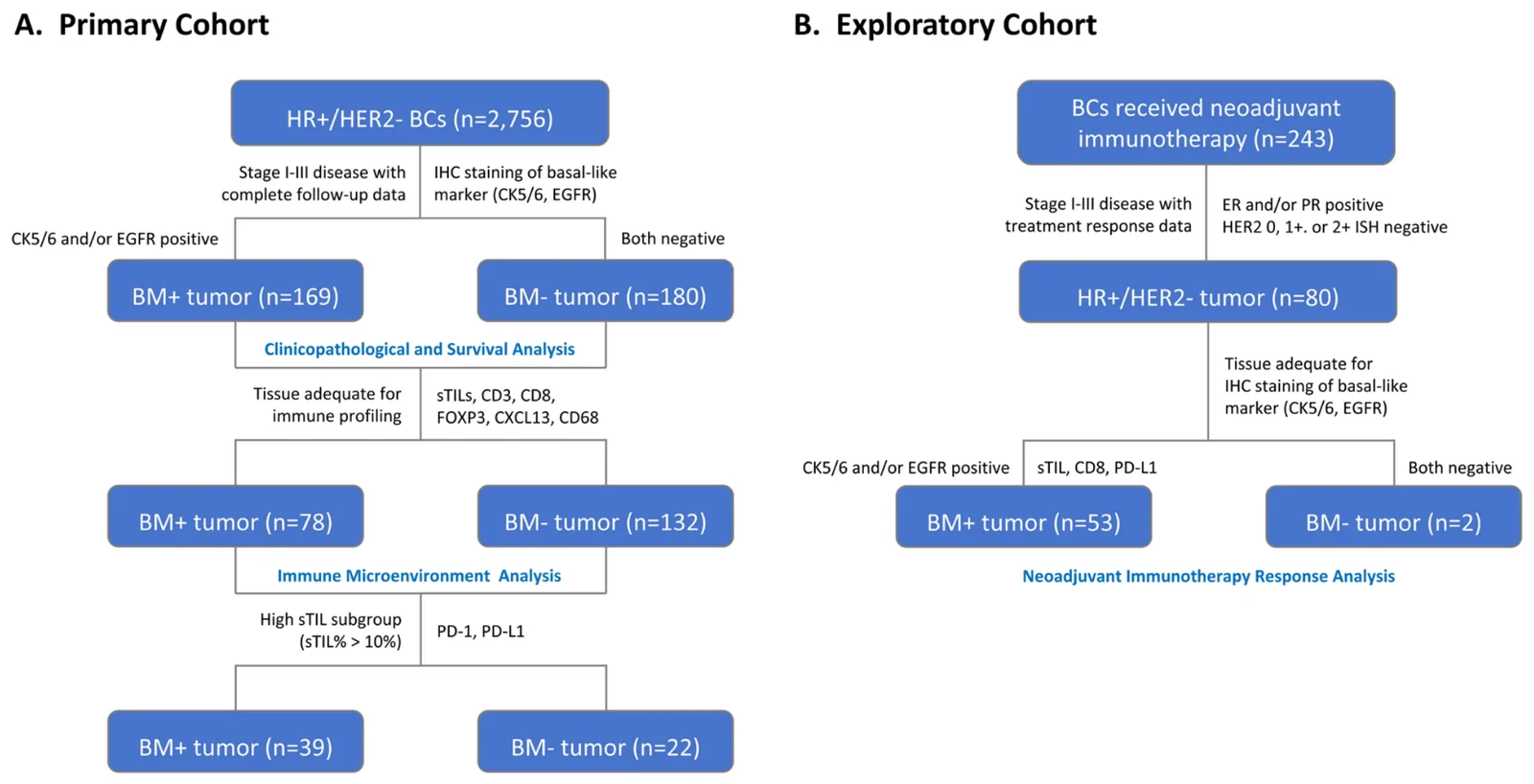
The flowchart of case selection in patient cohorts. (**A**) Primary cohort for clinicopathological features, immune microenvironment, and survival analysis. (**B**) Exploratory cohort for neoadjuvant immunotherapy response evaluation.

Patients diagnosed with HR+/HER2- invasive breast carcinoma of no special type (NST) at SYSUCC between January 2015 and December 2019 were identified from institutional databases (*n* = 2756). Basal marker status was determined by immunohistochemical (IHC) analysis of cytokeratin 5/6 (CK5/6, clone D5/16B4, Abcam) and epidermal growth factor receptor (EGFR, clone EP38Y, Abcam). Cases showing positive expression of at least one basal marker in ≥10% of tumor cells at any staining intensity constituted the BM+ group. After excluding patients with stage IV disease at diagnosis and those with incomplete follow-up data, 150 BM+ patients were included in the final analysis (Figure 1A). For comparative analysis, 180 BM- patients (negative for both CK5/6 and EGFR) with complete follow-up data were selected after propensity score matching from the same database and matched for clinical stage (AJCC 8th edition). Histopathological diagnosis and basal marker status were independently confirmed by two board-certified pathologists (JHZ, PS).

### 2.3. Exploratory Cohort: Neoadjuvant Immunotherapy Response

A separate cohort of patients who received neoadjuvant immunotherapy at SYSUCC between January 2020 and March 2024 was analyzed. From an initial pool of 243 BC patients treated with neoadjuvant chemotherapy combined with PD-1 inhibitor, 80 patients with stage I–III HR+/HER2- diseases were identified. After excluding cases with insufficient core needle biopsy tissue for basal marker assessment, 55 patients remained eligible. Among these, 53 patients (96.4%) demonstrated BM+ status and were included in the final analysis (Figure 1B). Treatment response was evaluated using the Miller–Payne grading system [15] and residual cancer burden (RCB) classification [16].

### 2.4. Clinicopathological Assessment and Survival Analysis

Comprehensive clinicopathological data were collected from institutional databases, including: age at diagnosis, tumor size, histological grade (Nottingham grading system), lymph node metastasis, lymphovascular invasion, presence of ductal carcinoma in situ (DCIS), calcification, fibrotic focus (FF) and tertiary lymphatic structures (TLSs), treatment modalities, and survival outcomes. Tumor stage was determined according to the 8th edition of the American Joint Committee on Cancer (AJCC) staging manual [17]. Hormone receptor status was assessed by IHC using standardized protocols: estrogen receptor (ER) and progesterone receptor (PR) were considered positive if ≥1% of tumor nuclei showed staining, following ASCO/CAP guidelines [18]. HER2 status was defined as negative for IHC scores of 0, 1+, or 2+ without gene amplification by fluorescence in situ hybridization (FISH) [19]. Ki-67 proliferation index was evaluated as the average percentage of positively stained tumor nuclei across representative tumor areas [20]. Survival endpoints included disease-free survival (DFS), defined as time from primary surgery to first recurrence (local, regional, or distant) or death from any cause, and overall survival (OS), defined as time from surgery to death from any cause.

### 2.5. Immune Microenvironment Characterization

Stromal tumor-infiltrating lymphocytes (sTILs) were evaluated on H&E-stained slides according to the international consensus guidelines established by the International Immuno-Oncology Biomarker Working Group in both cohorts [21]. sTILs were reported as the percentage of stromal area occupied by mononuclear inflammatory cells, excluding granulocytes and areas of crush artifact, necrosis, or intratumoral lymphocytes. Assessment was performed blinded to clinical outcomes. In the primary cohort, IHC staining was performed on representative FFPE tumor sections to characterize specific immune cell populations. The following antibodies were used: CD3 (clone LN10, ZSJQ-BIO) for total T cells, CD8 (clone OTI3H6, ZSJQ-BIO) for cytotoxic T cells, FOXP3 (clone 236A/E7, Abcam) for regulatory T cells, CD68 (clone PG-M1, ZSJQ-BIO) for macrophages, and CXCL13 (polyclonal, ZSJQ-BIO) for B-cell-attracting chemokine. Detailed antibody specifications and staining protocols are provided in Supplementary Table S1. Immune cell densities were quantified as the percentage of positive immune cells relative to total stromal cells within three representative high-power fields (×400 magnification). CXCL13 expression was assessed using a semiquantitative H-score system: H-score = (3 × percentage of strongly stained cells) + (2 × percentage of moderately stained cells) + (1 × percentage of weakly stained cells), yielding a range of 0–300. PD-1 (clone D4W2J, Cell Signaling Technology, Danvers, MA, USA) and PD-L1 (clone SP142, Abcam, Cambridge, CB, UK) expression was evaluated in a subset of 61 cases with sTILs > 10%. PD-1 positivity was defined as any membranous staining on immune cells, while PD-L1 positivity required ≥1% of immune cell area showing membranous staining of any intensity [22]. In the exploratory cohort, sTILs were evaluated on H&E-stained slides. IHC staining of CD8 (clone OTI3H6, ZSJQ-BIO) and PD-L1 (clone E1L3N, Cell Signaling Technology) was performed. PD-L1 was assessed using the Combined Positive Score (CPS), which is calculated as the number of PD-L1 staining cells (including tumor cells, lymphocytes, and macrophages) divided by the total number of viable tumor cells, and multiplied by 100 [23].

### 2.6. Statistical Analysis

Statistical analyses were performed using SPSS software (version 25.0, IBM Corporation). To reduce selection bias, we performed variable-ratio propensity score matching based on TNM stage, using a caliper width of 0.10. This yielded a final matched cohort of 150 BM+ and 180 BM- patients. Categorical variables were compared using chi-square or Fisher’s exact tests as appropriate. Correlations between basal marker expression and immune parameters were assessed using Pearson correlation coefficients. In the primary cohort, survival analyses were performed using the Kaplan–Meier method with log-rank tests for univariate comparisons. Multivariate Cox proportional hazards regression models were constructed to identify independent prognostic factors, with results expressed as hazard ratios (HRs) and 95% confidence intervals (CIs). In the exploratory cohort, logistic regression models were employed to analyze factors associated with pathological complete response (pCR), with results reported as odds ratios (ORs) and 95% confidence intervals (CIs). All statistical tests were two-sided, with *p* < 0.05 considered statistically significant.

## 3. Results

### 3.1. Aggressive Clinicopathological Features in HR+/HER2- BC with Basal-like Phenotype

**Figure 2:**
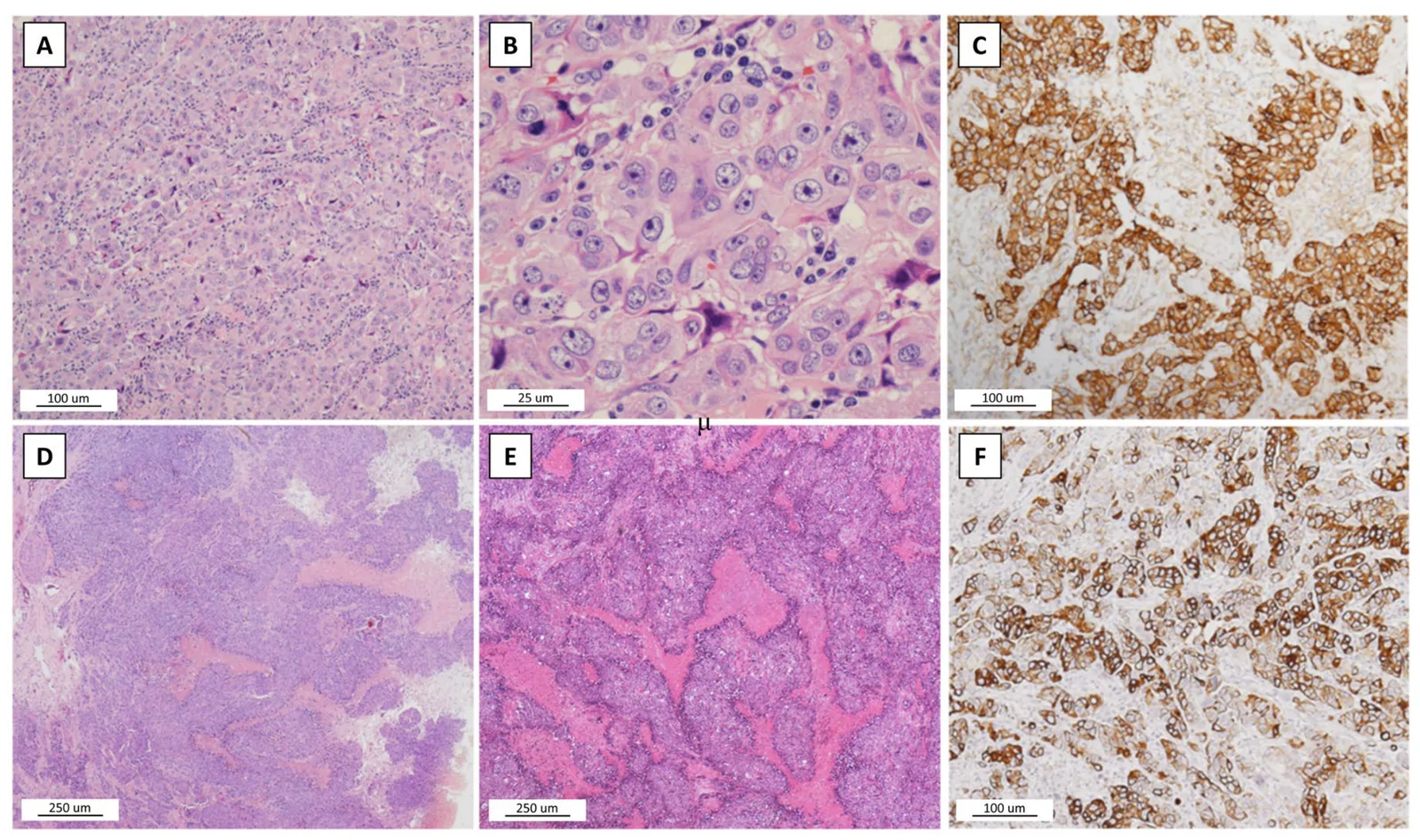
Morphological features of HR+/HER2- breast cancer with basal-like phenotype. Invasive breast cancer (BC) expressing basal markers (BMs) frequently comprises tumor cells with high histological grade, characterized by reduced or absent tubule formation (**A**) and marked nuclear pleomorphism (**B**). Necrosis is commonly observed in BM-positive BCs (**D**,**E**). The basal-like phenotype is characterized by typical strong and diffuse membrane/cytoplasmic staining of epidermal growth factor receptor (**C**) and/or cytokeratin 5/6 (**F**) in tumor cells.

Histomorphologically, BM+ tumors demonstrated characteristic basal-like features (Figure 2A), commonly showing solid sheet-like distribution of tumor cells with high nuclear grade and prominent nucleoli, minimal tubule formation, and obvious nuclear pleomorphism. Geographic necrosis, typically associated with triple-negative breast cancer, was more frequently observed in BM+ tumors. Consistent with the basal-like phenotype definition, biomarker analysis revealed uniform expression of basal markers in BM+ tumors, with 100% showing EGFR positivity and 52.0% exhibiting CK5/6 positivity (Figure 2B).

Our analysis revealed significant clinicopathological differences between BM+ and BM- HR+/HER2- breast cancer patients (Table 1). BM+ patients were diagnosed at a significantly younger age (median of 47 vs. 50 years, *p* = 0.031) and presented with larger tumor sizes (median of 2.5 vs. 2.0 cm, *p* = 0.024). Hormone receptor expression analysis revealed significant differences between groups. Low ER expression (≤50% positive) was observed in 61.3% of BM+ tumors, including 32.7% with ≤9% ER positivity and 28.6% with 10–50% ER positivity, compared to merely 1.7% (low ER expression, ≤50% positive) of BM- tumors (*p* < 0.001). Similarly, 82.7% of BM+ tumors demonstrated low PR expression (≤50% positive), comprising 59.4% with ≤9% PR positivity and 23.3% with 10–50% PR positivity, versus 33.9% (low PR expression, ≤50% positive) in BM- tumors (*p* < 0.001). The proliferation index Ki-67 was significantly elevated in BM+ tumors, with 40.7% showing Ki-67 > 50% compared to only 7.8% in BM- tumors (*p* < 0.001).

Regarding clinical management, the prevalence of chemotherapy in BM+ patients was more frequent than BM- patients (86.0% vs. 75.6%, *p* = 0.018), while the prevalence of endocrine therapy was administered less frequently in BM+ patients (74.0% vs. 87.2%, *p* = 0.002). No significant differences were observed between groups for surgical approaches (*p* = 0.218) or radiotherapy utilization (*p* = 0.883). As shown in Table 2, histopathological evaluation demonstrated that BM+ tumors exhibited more aggressive morphological features, including higher histological grade (grade III: 69.2% vs. 22.0%, *p* = 0.008) and increased tumor necrosis (26.9% vs. 10.6%, *p* = 0.002).

### 3.2. Prognostic Significance of Basal-like Phenotype in HR+/HER2- BC

**Figure 3:**
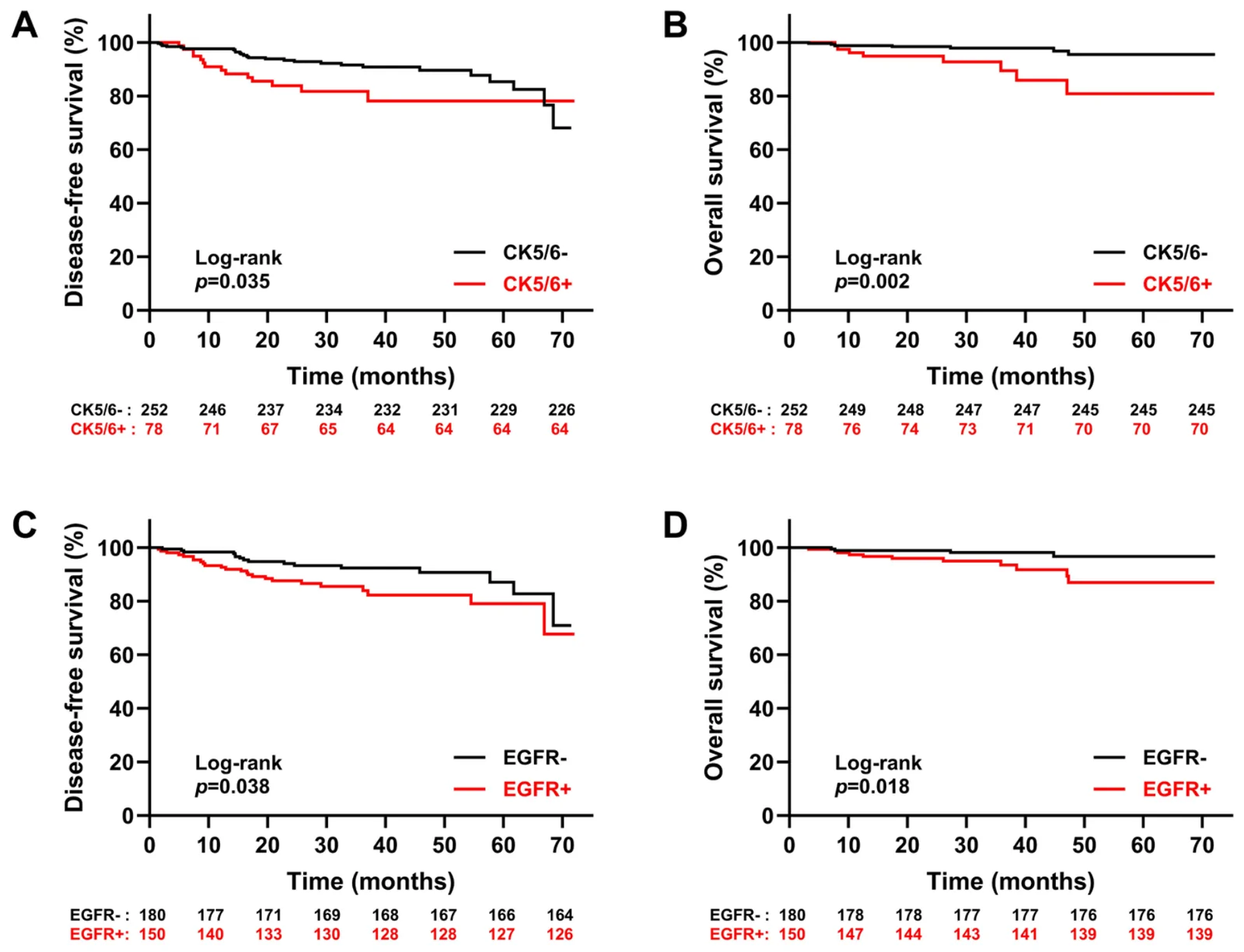
Kaplan–Meier survival curves for the impact of basal-like phenotype in HR+/HER2- breast cancer. (**A**,**B**) Impact of CK5/6 expression on disease-free survival (DFS) and overall survival (OS). (**C**,**D**) Impact of EGFR expression on DFS and OS.

Survival analysis demonstrated distinct prognostic impacts of basal markers in HR+/HER2- breast cancer. With a median follow-up of 34.42 months (range: 7.10–72.17 months) in HR+/HER2- patients, CK5/6 expression demonstrated robust independent prognostic value. Kaplan–Meier analysis revealed that CK5/6-positive patients exhibited significantly reduced DFS (Figure 3A, *p* = 0.035) and OS (Figure 3B, *p* = 0.002) compared to CK5/6-negative patients. Multivariate Cox regression analysis confirmed CK5/6 positivity as an independent predictor of poor outcomes for DFS (HR = 4.32, 95% CI: 1.60–11.61, *p* = 0.004) and OS (HR = 2.82, 95% CI: 1.44–5.55, *p* = 0.003) (Table 3). Similarly, patients with EGFR-positive tumors also showed significantly shorter DFS (Figure 3C, *p* = 0.038) and OS (Figure 3D, *p* = 0.018) compared to EGFR-negative patients, but this association did not remain significant in multivariate analysis. Additionally, clinical stage was demonstrated as an independent predictor for DFS (HR = 1.67, 95% CI: 1.27–2.20, *p* < 0.001) and OS (HR = 1.86, 95% CI: 1.55–2.24, *p* < 0.001).

### 3.3. Active Immune Microenvironment in HR+/HER2- BC with Basal-like Phenotype

**Figure 4:**
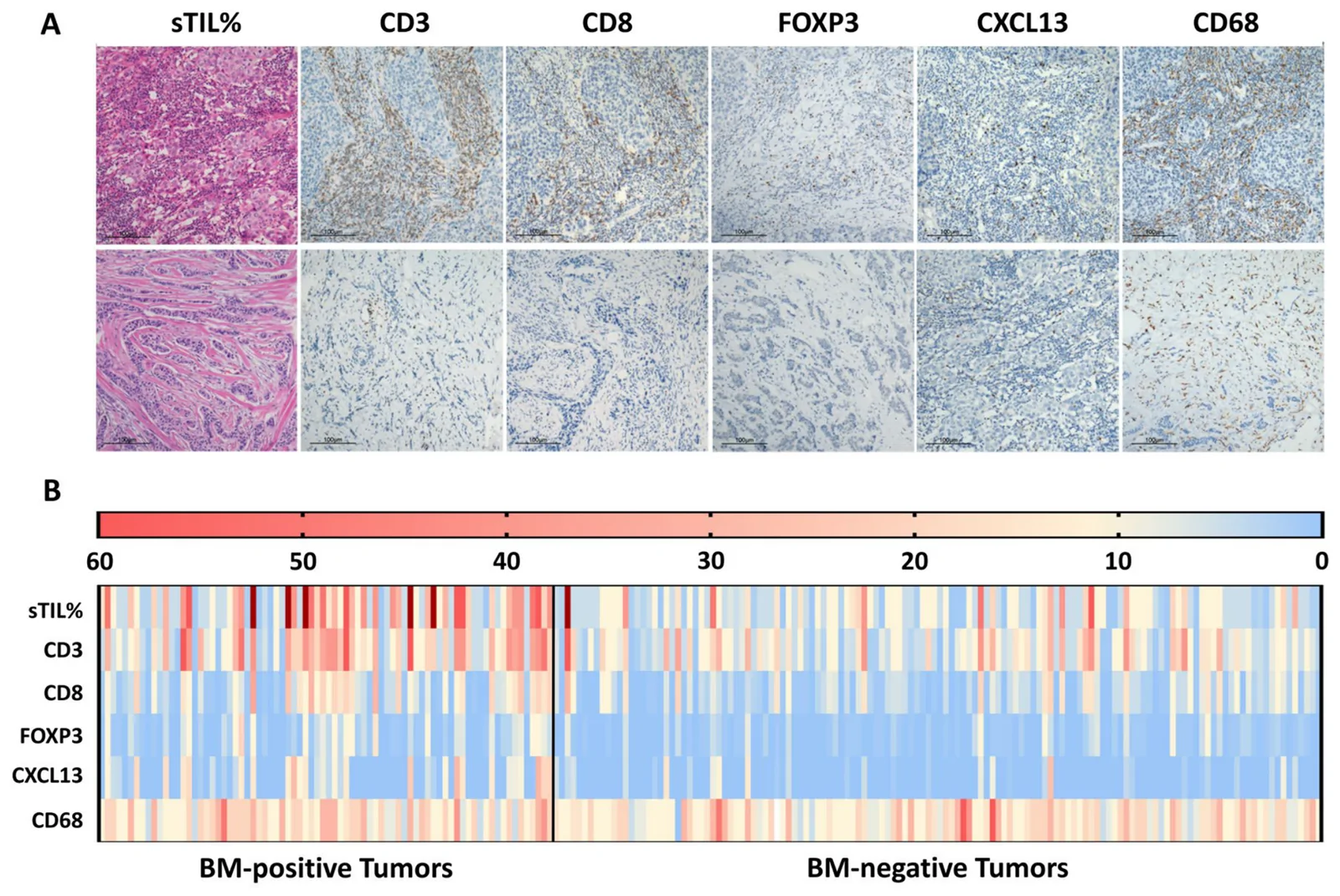
Comprehensive immune profiling and heatmap for the impact of basal-like phenotype in HR+/HER2- breast cancer. (**A**) Representative examples of high (**upper panels**) and low (**lower panels**) levels of stromal tumor-infiltrating lymphocytes (TILs) assessed on hematoxylin and eosin (H&E)-stained sections, along with specific immune cell populations evaluated by immunohistochemical (IHC) staining. (**B**) Expression profiles of immune biomarkers and corresponding heatmaps.

Comprehensive immune profiling revealed a significantly activated immune microenvironment in BM+ HR+/HER2- breast cancer (Table 4, Figure 4). Stromal TILs were substantially enriched in BM+ tumors, with 50.0% exhibiting high sTILs density compared to only 16.7% in BM- tumors (*p* < 0.001, correlation coefficient r = 0.36). This heightened lymphocytic infiltration extended to specific T-cell subsets: BM+ tumors showed significantly higher proportions of CD3+ T cells (52.6% vs. 28.0%, *p* < 0.001, r = 0.25), CD8+ cytotoxic T cells (51.3% vs. 30.3%, *p* = 0.002, r = 0.21), and FOXP3+ regulatory T cells (50.0% vs. 24.2%, *p* < 0.001, r = 0.26). Additionally, CXCL13 expression, a chemokine critical for B-cell recruitment and tertiary lymphoid structure formation, was significantly elevated in BM+ tumors (47.4% vs. 22.7%, *p* = 0.001, r = 0.26). Macrophage infiltration, as measured by CD68 expression, was also increased in BM+ tumors (48.7% vs. 34.1%, *p* = 0.036, r = 0.15).

Notably, within the subset of tumors harboring high sTILs (>10%), BM+ tumors demonstrated significantly higher rates of PD-1 positivity in immune cells (76.9% vs. 50.0%, *p* = 0.031, r = 0.28) and PD-L1 positivity (61.5% vs. 27.3%, *p* = 0.010, r = 0.33). These findings establish that HR+/HER2- breast cancers with basal-like phenotypes may exhibit a uniquely active immune microenvironment featuring enhanced lymphocyte infiltration, coordinated chemokine expression, and elevated immune checkpoint molecules, suggesting potential susceptibility to immunotherapy.

### 3.4. Neoadjuvant Immunotherapy Response in HR+/HER2- BC with Basal-like Phenotype

Given the immunologically active microenvironment observed in BM+ tumors, we evaluated the response to neoadjuvant immunotherapy in an independent exploratory cohort. This cohort represents a real-world population where clinicians selectively offered neoadjuvant immunotherapy to HR+/HER2- patients based on perceived high-risk features and potential immunotherapy benefit.

The exploratory cohort comprised predominantly high-risk patients with aggressive clinicopathological characteristics. As shown in Table 5, over half (54.7%) presented with stage III disease, and the majority displayed molecular features consistent with our primary cohort findings. Specifically, 75.5% exhibited low ER expression (<10% positive); 73.6% demonstrated low PR expression (<10% positive), and 73.6% had high proliferative activity (Ki-67 > 50%). Notably, 90.6% of the tumors were positive for both basal markers (EGFR and CK5/6), confirming the robust basal-like phenotype. The median stromal tumor-infiltrating lymphocytes (sTILs) density was 20%, with 81.1% of the cases classified as having high TILs. Similarly, 75.5% displayed high CD8+ T-cell infiltration (median 10%), and 52.8% had PD-L1 expression with CPS ≥ 10 (median CPS: 5), indicating an immune-activated tumor microenvironment.

All patients received neoadjuvant chemotherapy combined with PD-1 inhibitors, with different chemotherapy regimens distributed as follows: AC/EC-TCB (45.3%), AC/EC-T (22.6%), TCB (28.3%), and other regimens (3.8%). The pathological complete response (pCR) rate in this BM+ cohort was 49.1% (26/53). This pCR rate substantially exceeds the historical benchmark of 24–30% [12,13,14] typically observed in unselected HR+/HER2- breast cancer patients receiving neoadjuvant immunotherapy, and approaches the 58–66% [7,8,9,10,11,12] pCR rates commonly achieved in triple-negative breast cancer (TNBC). Among non-pCR patients, 11.3% achieved overall minimal residual disease (RCB-1), and 13.2% achieved minimal breast residual disease (Miller–Payne grade 4).

To identify independent predictors of treatment response, both univariate and multivariate logistic regression analyses were performed (Table 6). For the primary endpoint of pCR, the multivariate model revealed that younger age (OR, 0.78; 95% CI, 0.67–0.92; *p* = 0.002), higher levels of tumor-infiltrating lymphocytes (TILs) (OR, 1.12; 95% CI, 1.03–1.22; *p* = 0.010), and elevated PD-L1 expression (OR, 1.24; 95% CI, 1.06–1.44; *p* = 0.007) were significantly associated with an increased likelihood of achieving pCR. Although CD8+ T-cell infiltration showed a significant association in the univariate analysis, it did not retain independent significance in the multivariate model (OR, 0.86; 95% CI, 0.73–1.01; *p* = 0.074). In contrast, variables including cTNM stage, the specific chemotherapy regimen, standard tumor biomarkers (ER, PR, HER2, Ki-67), and basal markers (EGFR, CK5/6) showed no statistically significant predictive value for pCR. These results demonstrate that HR+/HER2- breast cancer with basal-like phenotype represents a distinct immunotherapy-responsive subset within the traditionally immunotherapy-resistant HR+/HER2- population, with response rates comparable to TNBC.

## 4. Discussion

The apparent resistance of HR+/HER2- breast cancer to immunotherapy has long been considered an immutable biological characteristic [13,14]. However, our study challenges this paradigm by demonstrating that this resistance may stem from our failure to recognize a distinct biological subset within this heterogeneous population. While basal-like markers (BMs) such as CK5/6 and EGFR have traditionally defined triple-negative breast cancer (TNBC) and are associated with poor prognosis [24,25,26,27], their expression in HR+/HER2- tumors identifies a clinically aggressive subgroup with unique therapeutic vulnerabilities that closely mirror TNBC biology [28].

Historically, the classification of breast cancer has relied primarily on hormone receptor and HER2 status, creating an artificial dichotomy that obscures significant biological diversity. Our findings reveal that BM-positive HR+/HER2- tumors represent a “hidden” basal-like phenotype that transcends conventional classification boundaries. These tumors exhibit remarkably low hormone receptor expressions, with 61.3% showing low ER expression and 82.7% demonstrating low PR expression, coupled with high proliferative activity and aggressive histopathological features, including frequent geographic necrosis. This molecular profile fundamentally challenges the notion that HR+/HER2- breast cancer constitutes a single disease entity and suggests that a substantial proportion of these tumors may actually represent misclassified basal-like cancers that retain minimal hormone receptor expression [29,30,31]. The combined detection of CK5/6 and EGFR has been shown to have predictive value in defining basal breast cancer [24,32], and EGFR represents a potential therapeutic target with available agents such as cetuximab and gefitinib [33].

The clinical implications of this biological reclassification are profound. Our survival analysis demonstrates that BM-positive status independently predicts poor outcomes in HR+/HER2- breast cancer, with CK5/6 expression emerging as a particularly robust prognostic marker. This poor prognosis cannot be adequately addressed by conventional adjuvant therapies, as traditional chemotherapy and endocrine therapy failed to mitigate the survival disadvantage observed in this subgroup. Even in certain invasive subtypes of breast cancer, surgical interventions such as salvage mastectomy do not appear to improve patient prognosis [34]. However, the very features that confer aggressiveness also create a therapeutic opportunity: BM-positive tumors exhibit a uniquely activated immune microenvironment characterized by significantly higher stromal tumor-infiltrating lymphocytes (50.0% vs. 16.7%), enhanced cytotoxic T-cell infiltration, elevated CXCL13 expression, and increased PD-1/PD-L1 checkpoint molecule expression [35,36]. This immune-hot phenotype stands in stark contrast to the typically immunologically cold nature of luminal breast cancer and provides the biological rationale for immunotherapy intervention. The tumor immune microenvironment (TIME), after complex transformation from an initial inflammatory reaction, is associated with immune escape and tumor progression [37], though increased TILs have been reported as an adverse predictive factor for neoadjuvant therapy response in ER+/HER2- tumors [38].

The transformative potential of this insight is validated by our exploratory cohort evaluating neoadjuvant immunotherapy response. Among BM-positive HR+/HER2- patients, the pathological complete response rate reached 49.1%—a figure that dramatically exceeds the historical 24–30% benchmark for unselected HR+/HER2- patients [12,13,14] and approaches the 58–66% rates achieved in TNBC [7,8,9,10,11,12]. Pembrolizumab, a PD-1 inhibitor, has received FDA approval for high-risk early-stage triple-negative breast cancer in the neoadjuvant setting combined with chemotherapy, followed by continued adjuvant treatment [8,39], and our data suggest similar benefit may extend to this biologically defined HR+/HER2- subset. This remarkable response rate provides compelling evidence that the apparent immunotherapy resistance of HR+/HER2- breast cancer is not intrinsic to the entire subtype but rather reflects the inclusion of patients who lack the necessary immunological prerequisites for response. By using simple, clinically accessible IHC markers (CK5/6 and EGFR), we can identify patients whose tumors biologically resemble TNBC and who derive exceptional benefit from immunotherapy combinations. When clinicians evaluate whether immunotherapy should be administered to HR+/HER2- breast cancer patients, the BM status, particularly CK5/6-positive or not, may enable more effective screening for a part of HR+/HER2- breast cancer patients who are sensitive to immunotherapy, thereby assisting clinicians in making more informed clinical decisions. This approach aligns with the precision medicine paradigm by matching therapeutic interventions to underlying tumor biology rather than relying solely on receptor status. The identification of younger age, high TILs, and elevated PD-L1 as independent predictors of response further refines patient selection within the BM-positive subgroup.

Our study has limitations, including its retrospective, single-institution design with potential missing information, limited tissue for comprehensive TIME analysis, and heterogeneous adjuvant treatment regimens. Since BM-negative HR+/HER2- breast cancer cases are exceedingly rare in real-world settings that adopt this neoadjuvant therapy regimen, our neoadjuvant immunotherapy cohort lacked complete control groups, specifically BM-negative HR+/HER2- and BM-positive TNBC patients, to definitively validate the magnitude of immunotherapy benefit. In addition, our multivariate analysis (Table 6) also revealed associations between age, TILs, and PD-L1 expression. Furthermore, we cannot rule out the possibility that the relatively high pCR rate may be attributed to low HR expression, high Ki-67 levels, or the choice of chemotherapy regimen. Nevertheless, the consistency between our pathological, immunological, and clinical response data provides robust evidence that basal marker expression identifies a clinically aggressive HR+/HER2- subset requiring novel therapeutic strategies. Future prospective trials with standardized protocols and appropriate control groups should validate whether BM-positive patients benefit from TNBC-like treatment algorithms incorporating immunotherapy. The clinical impact of this approach could be substantial, potentially transforming outcomes for a high-risk subgroup that currently lacks effective therapeutic options.

## 5. Conclusions

The basal-like phenotype identifies a clinically aggressive subset of HR+/HER2- breast cancer with distinct immune-activated characteristics and remarkable immunotherapy response rates. These findings suggest that the BM status, particularly CK5/6 expression, may serve as a basis for clinicians to decide whether to administer immunotherapy to patients with HR+/HER2- breast cancer.

## Figures and Tables

**Table 1:** Clinical features of HR+/HER2- BC with basal-like phenotype in primary cohort.

Characteristics	Total (*n* = 330)	BM+ (*n* = 150)	CK5/6+ (*n* = 78)	BM- (*n* = 180)	*p* *	*p* **
Age at diagnosis						
Median age, years	48	47	45	50	0.031	<0.001
<40	49 (14.8)	25 (16.7)	20 (25.7)	24 (13.3)	0.067	0.025
40–55	194 (58.8)	93 (62.0)	43 (55.1)	101 (56.1)		
>55	87 (26.4)	32 (21.3)	15 (19.2)	55 (30.6)		
Tumor size (pT)					0.061	0.004
pT1	152 (46.1)	58 (38.7)	23 (29.5)	94 (52.2)		
pT2	156 (47.3)	85 (56.7)	50 (64.1)	71 (39.4)		
pT3	17 (5.2)	5 (3.3)	4 (5.1)	12 (6.7)		
pT4	5 (1.5)	2 (1.3)	1 (1.3)	3 (1.7)		
Lymphatic metastasis (pN)					0.412	0.112
pN0	174 (52.7)	83 (55.3)	45 (57.7)	91 (50.6)		
pN1	94 (28.5)	42 (28.0)	23 (29.5)	52 (28.9)		
pN2	35 (10.6)	10 (6.7)	3 (3.8)	25 (13.9)		
pN3	27 (8.2)	15 (10.0)	7 (9.0)	12 (6.7)		
pTNM staging					0.536	0.011
I	88 (26.7)	34 (22.7)	14 (17.9)	54 (30.0)		
IIa	131 (39.7)	67 (44.7)	39 (50.0)	64 (35.6)		
IIb	47 (14.2)	23 (15.3)	14 (17.9)	24 (13.3)		
IIIa	35 (10.6)	9 (6.0)	3 (3.9)	26 (14.4)		
IIIb	2 (0.6)	2 (1.3)	0	0		
IIIc	27 (8.2)	15 (10.0)	8 (10.3)	12 (6.7)		
ER					<0.001	<0.001
<10%	49 (14.9)	49 (32.7)	41 (52.6)	0		
10–50%	46 (13.9)	43 (28.6)	28 (35.9)	3 (1.7)		
>50%	235 (71.2)	58 (38.7)	9 (11.5)	177 (98.3)		
PR					<0.001	<0.001
<10%	114 (34.5)	89 (59.4)	61 (78.3)	25 (13.9)		
10–50%	71 (21.6)	35 (23.3)	14 (17.9)	36 (20.0)		
>50%	145 (43.9)	26 (17.3)	3 (3.8)	119 (66.1)		
HER2					0.359	0.213
0	209 (63.3)	99 (66.0)	54 (69.2)	110 (61.1)		
1+/2+	121 (36.7)	51 (34.0)	24 (30.8)	70 (38.9)		
Ki-67					<0.001	<0.001
<15%	81 (24.5)	20 (13.3)	2 (2.6)	61 (33.9)		
15–50%	174 (52.7)	69 (46.0)	26 (33.3)	105 (58.3)		
>50%	75 (22.7)	61 (40.7)	50 (64.1)	14 (7.8)		
BM status					-	-
Both positive	78 (23.6)	78 (52)	0	0 (0)		
Only EGFR-positive	150 (45.5)	150 (100)	0	0 (0)		
Only CK5/6-positive	0 (0)	0 (0)	78 (100)	0 (0)		
Surgery					0.218	0.865
Breast conservation	53 (16.1)	20 (13.3)	15 (19.2)	33 (18.3)		
Mastectomy	277 (83.9)	130 (86.7)	63 (80.8)	147 (81.7)		
Chemotherapy					0.018	0.167
Yes	265 (80.3)	129 (86.0)	65 (83.3)	136 (75.6)		
No	65 (19.7)	21 (14.0)	13 (16.7)	44 (24.4)		
Endocrine therapy					0.002	<0.001
Yes	268 (81.2)	111 (74.0)	50 (64.1)	157 (87.2)		
No	62 (18.8)	39 (26.0)	28 (35.9)	23 (12.8)		
Radiotherapy					0.883	0.870
Yes	118 (35.8)	53 (35.3)	29 (37.2)	65 (36.1)		
No	212 (64.2)	97 (64.7)	49 (62.8)	115 (63.9)		
Local relapse					1.000	1.000
Yes	2 (0.6)	1 (0.7)	0	1 (0.6)		
No	328 (99.4)	149 (99.3)	78 (100)	179 (99.4)		
Distant metastasis					0.047	0.025
Yes	38 (11.5)	23 (15.3)	14 (17.9)	15 (8.3)		
No	292 (88.5)	127 (84.7)	64 (82.1)	165 (91.7)		
Death					0.055	0.025
Yes	16 (4.8)	11 (7.3)	8 (10.3)	5 (2.8)		
No	314 (95.2)	139 (92.7)	70 (89.7)	175 (97.2)		

BM, basal-like marker; HR, hormone receptor; BC, breast cancer; ER, estrogen receptor; PR, progesterone receptor; HER2, human epidermal growth factor receptor 2; pT, tumor size; pN, lymphatic metastasis. * χ2 test comparing proportions among BM+ group vs. BM- group. ** χ2 test comparing proportions among CK5/6+ group vs. BM- group.

**Table 2:** Association of BM expression with pathological features in HR+/HER2- BC.

Characteristics	Total (*n* = 210)	BM+ (*n* = 78)	CK5/6+ (*n* = 32)	BM- (*n* = 132)	*p* *	*p* **
Histological grade					0.008	<0.001
I	3 (1.4)	0	0	3 (2.3)		
II	124 (59.0)	24 (30.8)	4 (12.5)	100 (75.8)		
III	83 (39.5)	54 (69.2)	28 (87.5)	29 (22.0)		
DCIS					0.050	0.016
Yes	91 (43.3)	27 (34.6)	8 (25.0)	64 (48.5)		
No	119 (56.7)	51 (65.4)	24 (75.0)	68 (51.5)		
LVI					0.384	0.196
Yes	73 (34.8)	24 (30.8)	8 (25.0)	49 (37.1)		
No	137 (65.2)	54 (69.2)	24 (75.0)	83 (62.9)		
Necrosis					0.002	<0.001
Yes	35 (16.7)	21 (26.9)	13 (40.6)	14 (10.6)		
No	175 (83.3)	57 (73.1)	19 (59.4)	118 (89.4)		
Calcification					0.069	0.734
Yes	31 (14.8)	7 (9.0)	5 (15.6)	24 (18.2)		
No	179 (85.2)	71 (91.0)	27 (84.4)	108 (81.8)		
Fibrotic focus					0.923	0.559
Yes	37 (17.6)	14 (17.9)	7 (21.9)	23 (17.4)		
No	173 (82.4)	64 (82.1)	25 (78.1)	109 (82.6)		
TLS					0.750	0.384
Yes	7 (3.3)	3 (3.8)	2 (6.2)	4 (3.0)		
No	203 (96.7)	75 (96.2)	30 (93.8)	128 (97.0)		

BM, basal-like marker; HR, hormone receptor; BC, breast cancer; DCIS, ductal carcinoma in situ; LVI, lymphovascular invasion; TLS, tertiary lymphatic structure. * χ2 test comparing proportions among BM+ group vs. BM- group. ** χ2 test comparing proportions among CK5/6+ group vs. BM- group.

**Table 3:** Univariate and multivariate analyses of prognostic factors for DFS and OS.

	Univariate Analysis *			Multivariate Analysis		
	HR	95% CI	*p*	HR	95% CI	*p*
**DFS**						
pTNM stage	1.62	1.24–2.13	0.001	1.67	1.27–2.20	<0.001
CK5/6 (pos. vs. neg.)	3.88	1.45–10.39	0.007	4.32	1.60–11.61	0.004
EGFR (pos. vs. neg.)	2.93	1.02–8.45	0.047	-	-	0.580
ER	0.97	0.97–1.00	0.026	-	-	0.990
PR	0.98	0.96–0.99	0.010	-	-	0.122
Ki-67	1.02	1.00–1.04	0.016	-	-	0.585
**OS**						
pTNM stage	1.81	1.51–2.15	<0.001	1.86	1.55–2.24	<0.001
CK5/6 (pos. vs. neg.)	2.03	1.06–3.90	0.033	2.82	1.44–5.55	0.003
EGFR (pos. vs. neg.)	1.96	1.04–3.69	0.038	-	-	0.314
PR	0.99	0.98–1.00	0.025	-	-	0.175
Ki-67	1.02	1.01–1.03	0.006	-	-	0.218
Chemotherapy (yes vs. no)	4.85	1.17–20.11	0.030	-	-	0.110
Radiation (yes vs. no)	2.86	1.51–5.43	0.001	-	-	0.055

DFS, disease-free survival; OS, overall survival; ER, estrogen receptor; PR, progesterone receptor; HR, hazard ratio; CI, confidence interval. * Univariate analysis included age, pTNM stage, ER, PR, HER2, Ki67 expression, basal markers (EGFR, CK5/6) status, and clinical treatment. Only factors that demonstrated statistical significance in the univariate analysis were subsequently included in the multivariate model.

**Table 4:** Association between basal-like phenotype and immune biomarkers in HR+/HER2- BC.

Immune Biomarkers *	Total	BM+	BM-	Correlation Coefficient	*p*
TILs				0.36	<0.001
Low	149 (71.0)	39 (50.0)	110 (83.3)		
High	61 (29.0)	39 (50.0)	22 (16.7)		
CD3				0.25	<0.001
Low	132 (62.9)	37 (47.4)	95 (72.0)		
High	78 (37.1)	41 (52.6)	87 (28.0)		
CD8				0.21	0.002
Low	130 (61.9)	38 (48.7)	92 (69.7)		
High	80 (38.1)	40 (51.3)	40 (30.3)		
FOXP3				0.26	<0.001
Low	139 (66.2)	39 (50.0)	100 (75.8)		
High	71 (33.8)	39 (50.0)	32 (24.2)		
CXCL13				0.26	0.001
Low	143 (68.1)	41 (52.6)	102 (77.3)		
High	67 (31.9)	37 (47.4)	30 (22.7)		
CD68				0.15	0.036
Low	127 (60.5)	40 (51.3)	87 (65.9)		
High	83 (39.5)	38 (48.7)	45 (34.1)		
PD-1				0.28	0.031
Positive	41 (67.2)	30 (76.9)	11 (50.0)		
Negative	20 (32.8)	9 (23.1)	11 (50.0)		
PD-L1				0.33	0.010
Positive	30 (49.2)	24 (61.5)	6 (27.3)		
Negative	31 (50.8)	15 (38.5)	16 (72.7)		

BM, basal-like marker; HR, hormone receptor; BC, breast cancer; TILs, tumor-infiltrating lymphocytes; PD-1, programmed cell death protein 1; PD-L1: programmed death-ligand 1. * Immune biomarker status, including sTILs%, CD3, CD8, FOXP3, and CXCL13 expression, was assessed in 210 cases. PD-1 and PD-L1 expressions were evaluated in a subset of 61 cases with stromal TILs >10%.

**Table 5:** Clinicopathological features of HR+/HER2- BC with basal-like phenotype in exploratory cohort.

Characteristics	BM+ (*n* = 53)
Age at diagnosis	
Median age, years	47
<40	11 (20.8)
40–55	29 (54.7)
>55	13 (24.5)
cT	
cT1	7 (13.2)
cT2	30 (56.6)
cT3	11 (20.8)
cT4	5 (9.4)
cN	
cN0	7 (13.2)
cN1	22 (41.5)
cN2	11 (20.8)
cN3	13 (24.5)
cTNM staging	
I	2 (3.8)
IIa	6 (11.3)
IIb	16 (30.2)
IIIa	12 (22.7)
IIIb	4 (7.5)
IIIc	13 (24.5)
ER	
<10%	40 (75.5)
10–50%	11 (20.8)
>50%	2 (3.7)
PR	
<10%	39 (73.6)
10–50%	13 (24.5)
>50%	1 (1.9)
HER2	
0	23 (43.4)
1+/2+	30 (56.7)
Ki-67	
<15%	0
15–50%	14 (26.4)
>50%	39 (73.6)
BM status	
Both positive	48 (90.6)
Only EGFR-positive	4 (7.5)
Only CK5/6-positive	1 (1.9)
TILs	
Median, %	20
Low	10 (18.9)
High	43 (81.1)
CD8	
Median, %	10
Low	13 (24.5)
High	40 (75.5)
PD-L1 (22C3)	
Median, CPS	5
CPS ≥ 10	28 (52.8)
CPS < 10	25 (47.2)
Chemotherapy	
AC/EC→TCB + PD1 inhibitor *	24 (45.3)
AC/EC→T + PD1 inhibitor *	12 (22.6)
TCB + PD1 inhibitor *	15 (28.3)
Other + PD1 inhibitor *	2 (3.8)
Treatment response	
pCR	26 (49.1)
Non-pCR	27 (50.9)
RCB grade	
RCB-0	26 (49.1)
RCB-1	6 (11.3)
RCB-2	12 (22.6)
RCB-3	9 (17.0)
Miller–Payne grade	
G5	26 (49.1)
G4	7 (13.2)
G3	11 (20.8)
G2	6 (11.3)
G1	3 (5.6)

BM, basal-like marker; HR, hormone receptor; BC, breast cancer; ER, estrogen receptor; PR, progesterone receptor; HER2, human epidermal growth factor receptor 2; TILs, tumor-infiltrating lymphocytes; PD-L1, programmed death-ligand 1; CPS, Combined Positive Score; AC, doxorubicin + cyclophosphamide; EC, epirubicin + cyclophosphamide; TCB, paclitaxel + carboplatin; T, paclitaxel; PD-1, programmed cell death protein 1; pCR, pathological complete response; RCB, residual cancer burden. * PD-1 inhibitors: tremelimumab or pembrolizumab.

**Table 6:** Univariate and multivariate analyses of neoadjuvant immunotherapy response in HR+/HER2- BC with basal-like phenotype.

	Univariate Analysis *			Multivariate Analysis		
	OR	95% CI	*p*	OR	95% CI	*p*
pCR						
Age	0.91	0.85–0.97	0.006	0.78	0.67–0.92	0.002
TILs	1.06	1.02–1.11	0.004	1.12	1.03–1.22	0.010
CD8	1.10	1.03–1.17	0.007	-	-	0.074
PD-L1	1.09	1.02–1.16	0.009	1.24	1.06–1.44	0.007

HR, hormone receptor; BC, breast cancer; HER2, human epidermal growth factor receptor 2; pCR, pathological complete response; TILs, tumor-infiltrating lymphocytes; PD-L1, programmed death-ligand 1; OR, odds ratio; CI, confidence interval. * Univariate analysis included age, cTNM stage, ER, PR, HER2, and Ki67 expressions, basal markers (EGFR, CK5/6), immune microenvironment makers (TILs, CD8, PD-L1) and chemotherapy regimen. Only factors that demonstrated statistical significance in the univariate analysis were subsequently included in the multivariate model.

## Data Availability

The datasets supporting the conclusions of this article are available from the corresponding authors upon reasonable request.

## Supplementary Materials

The following supporting information can be downloaded at https://www.mdpi.com/article/10.3390/cancers18182965/s1, Supplementary Table S1. Details of antibodies and experimental conditions in this study.

## Abbreviations

The following abbreviations are used in this manuscript:

BC	Breast cancer
BM	Basal-like marker
BM+	Basal marker-positive
BM-	Basal marker-negative
CD	Cluster of differentiation
CI	Confidence interval
CK5/6	Cytokeratin 5/6
CPS	Combined Positive Score
CXCL13	(C-X-C motif) ligand 13
DCIS	Ductal carcinoma in situ
DFS	Disease-free survival
EGFR	Epidermal growth factor receptor
ER	Estrogen receptor
FF	Fibrotic focus
FISH	Fluorescence in situ hybridization
FOXP3	Forkhead box P3
HER2	Human epidermal growth factor receptor 2
HR+	Hormone receptor-positive
HR	Hazard ratio
IC	Immune cell
IHC	Immunohistochemistry
LVI	Lymphovascular invasion
NST	No special type
OR	Odds ratio
OS	Overall survival
PAM50	Prediction Analysis of Microarray 50
pCR	Pathological complete response
PD-1	Programmed cell death protein 1
PD-L1	Programmed cell death-ligand 1
PR	Progesterone receptor
RCB	Residual cancer burden
sTIL	Stromal tumor-infiltrating lymphocyte
TIL	Tumor-infiltrating lymphocyte
TIME	Tumor immune microenvironment
TLS	Tertiary lymphatic structure
TNBC	Triple-negative breast cancer
TNP	Triple-negative phenotype
