# Supplementary material for: Basal-like Phenotype Identifies Immunotherapy-Responsive Subset of HR+/HER2- Breast Cancer with Aggressive Clinical Behavior
Source: Cancers (Basel). 2026 Sep 14;18(18):2965. doi: 10.3390/cancers18182965 (PMC13604159; doi:10.3390/cancers18182965)
Supplement: Supplementary file 1 [file cancers-18-02965-s001.zip › cancers-4464738-supplementary.pdf]

Supplementary Table S1. Details of antibodies and experimental conditions in this study.

Antibodies	Company	Reference	Clone	Origin	Isotype	Antigen Retrieval	Dilution
EGFR	Abcam, Cambridge, UK	ab52894	EP38Y	Monoclonal rabbit	IgG	Tris-EDTA pH9.0	1:100
CK5/6	Abcam, Cambridge, UK	ab17133	D5/16 B4	Monoclonal mouse	IgG1	Tris-EDTA pH8.0	1:100
CD3	ZSJQ-BIO, Beijing, China	ZM-0417	LN10	Monoclonal mouse	IgG1	Tris-EDTA pH8.0	1:100
CD8	ZSJQ-BIO, Beijing, China	TA802079	OTI3H6	Monoclonal mouse	IgG1	Tris-EDTA pH9.0	1:800
FOXP3	Abcam, Cambridge, UK	ab20034	236A/E7	Monoclonal mouse	IgG	Tris-EDTA pH9.0	1:400
CXCL13	ZSJQ-BIO, Beijing, China	ZG-0601	-	Polyclonal goat	IgG	Tris-EDTA pH9.0	prediluted
CD68	ZSJQ-BIO, Beijing, China	ZM-0464	PG-M1	Monoclonal mouse	IgG3	Tris-EDTA pH9.0	1:800
PD-1	Cell Signaling Tech, Boston, USA	86163T	D4W2J	Monoclonal rabbit	IgG	Tris-EDTA pH9.0	1:200
PD-L1	Abcam, Cambridge, UK	ab228462	SP142	Monoclonal rabbit	IgG	Tris-EDTA pH9.0	1:400
